# A Lifespan Perspective on Embodied Cognition

**DOI:** 10.3389/fpsyg.2016.00845

**Published:** 2016-06-01

**Authors:** Jonna Loeffler, Markus Raab, Rouwen Cañal-Bruland

**Affiliations:** ^1^Department of Performance Psychology, Institute of Psychology, German Sport University CologneCologne, Germany; ^2^School of Applied Sciences, London South Bank UniversityLondon, UK; ^3^Department of Human Movement Sciences, Vrije Universiteit AmsterdamAmsterdam, Netherlands

**Keywords:** embodiment, lifespan, developmental, elderly, sensorimotor, cognition

## Abstract

Since its infancy embodied cognition research has fundamentally changed our understanding of how action, perception, and cognition relate to and interact with each other. Ideas from different schools of thought have led to controversial theories and a unifying framework is still being debated. In this perspective paper, we argue that in order to improve our understanding of embodied cognition and to take significant steps toward a comprehensive framework, a lifespan approach is mandatory. Given that most established theories have been developed and tested in the adult population, which is characterized by relatively robust and stable sensorimotor and cognitive abilities, we deem it questionable whether embodied cognition effects found in this population are representative for different life stages such as childhood or the elderly. In contrast to adulthood, childhood is accompanied by a rapid increase of sensorimotor and cognitive skills, and the old age by a decline of such capacities. Hence, sensorimotor and cognitive capacities, as well as their interactions, are more fragile at both extremes of the lifespan, thereby offering a unique window into the emergence of embodied cognition effects and age-related differences therein. A lifespan approach promises to make a major contribution toward a unifying and comprehensive theory of embodied cognition that is valid across the lifespan and ‘gets better with age.’

## Introduction

For a long time, cognition was considered to reflect an internal process, with information being received, organized, and retrieved by the mind (Fodor, 1975). For instance, problem-solving was thought to be entirely explained as a mental process of activating and combining prior knowledge (Newell and Simon, 1972). This approach, known as cognitivism, was dominant in the study of cognition until the early 1990s when embodied cognition research departed from traditional views and put forth the idea that sensorimotor processes have an undeniable influence on cognition (Varela et al., 1992).

In contrast to cognitivism, embodied cognition views argue that sensorimotor processes are at the core of human cognitive functioning. Sensorimotor processes, in a general sense, refer to and include any processes that give rise to action such as, for example, sensorimotor coordination and sensorimotor contingencies (Engel et al., 2013). To remain with the example of problem-solving: recently, Werner and Raab (2014) showed that sensorimotor activities, in form of whole-body movements, directly impact problem-solving strategies, thereby providing further evidence for a tight link between sensorimotor and cognitive processes. On the one hand, embodied cognition research successfully enriched empirical work on numerous cognitive functions such as language (Casasanto, 2011), memory (Versace et al., 2014), and attention (Bradley, 2007); on the other hand, there are several theoretical accounts that are currently debated but that have not led to a comprehensive theoretical framework yet. A recent proposal for such a comprising framework was submitted by Gentsch et al. (2016, p. 88): they propose a meta-theoretical framework referred to as “grounded action cognition” that aims to accommodate three main families of embodied cognition accounts: common coding, internal models, and simulation theories. Additionally, more radical accounts on embodied cognition (e.g., O’Regan and Noë, 2001; Chemero, 2011) suggest that perception is a form of action. The chief commonality between these embodied cognition theories (which at the same time served as the inclusion criterion for the studies included in this perspective paper) is that they assume an undeniable influence of sensorimotor processes on cognition (for an overview, see also Fischer and Coello, 2016). The aim of this perspective paper is not to discuss the individual theoretical contributions and core assumptions of either theory. Rather, we are concerned that perhaps none of the mentioned theoretical accounts may be able to provide a comprehensive framework that is valid across the lifespan. The above-mentioned candidate theories of embodied cognition, at large, were developed based on data acquired in the adult population and, therefore, run the risk of being overgeneralized if conclusions were to be drawn or predictions generated for the extremes of the lifespan continuum.

In keeping with Vallet (2015), who focused on the impact of embodied cognition on aging and neurocognitive disorders, we argue that a lifespan approach is mandatory, because current embodied cognition theories have not been validated across the lifespan and may, therefore, only be representative for the adult populations put at test. Because adulthood is characterized by relatively robust and stable sensorimotor and cognitive abilities, it is questionable whether embodied cognition effects found in this population are representative for the whole age spectrum. Across the lifespan humans undergo manifold developmental changes including developing and degenerating sensorimotor and cognitive abilities. Childhood is accompanied by individually varying increases of sensorimotor and cognitive skills, that is, children learn cognitive as well as sensorimotor skills relatively fast. They traverse various developmental stages and constantly make novel, exploratory experiences with their environments (Daum et al., 2009), thereby accumulating new sensorimotor contingencies (O’Regan and Noë, 2001). For elderly people learning cognitive as well as sensorimotor skills becomes increasingly difficult; for instance, their sensorimotor and cognitive flexibility decreases (Shephard et al., 1990; Greenwood, 2007). Yet, elderly people have also accumulated more experience, including sensorimotor experience and possess increased knowledge (e.g., Blanco et al., 2016). Therefore, sensorimotor and cognitive functions during childhood and in the elderly differ in various ways.

Given these differences at the extremes of the lifespan continuum, there is surprisingly little research examining the predictions of embodied cognition accounts across the different age groups. In addition, the accumulated evidence on embodied cognition effects that included age as a factor reports rather mixed findings and is, therefore, inconclusive (e.g., Dijkstra et al., 2007; Frick et al., 2009). To allow for a comparison between embodied cognition studies across the lifespan, a promising route to structure the available literature is to specify the processes underlying embodied cognition effects. We suggest that these processes can be categorized as driven by either *new* or *reactivated* associations (for similar classifications, see Craik and Bialystok, 2006; Shing and Lindenberger, 2011). In the paper at hand, we refer to associations as connections between conceptual entities or sensorimotor/cognitive states that derive from a similarity between those states or their proximity in space or time (Mifflin, 2001). At the same time, this definition implies concomitant processes on the neurological level like synaptic associations or firing of populations of neurons. In a nutshell, a sensorimotor change (e.g., a movement) can create *new* multimodal associations the moment it is executed. On the other hand, a sensorimotor change can *reactivate* previous associations. This type of embodied cognition effect is driven by previously generated associations and is, therefore, experience-based and memory-driven in nature. The rationale behind this categorization is that the likelihood for interference between new and reactivated associations is reduced to a minimum at the beginning of life, whereas in the later stages of life the impact of reactivated associations may reach their maximum.

To reiterate, a lifespan approach promises to make a major contribution to theory formation in the field of embodied cognition. To sketch out the importance of a lifespan approach and to offer promising avenues for future research we critically discuss evidence for embodied cognition effects and their underlying mechanisms with respect to *new* versus *reactivated* associations, in three steps. First, we focus on embodied cognition effects in infants; second, we discuss embodied cognition effects in the elderly; and third, we examine the very few studies that have actually compared embodied cognition effects between age groups.

## Embodied Cognition Effects in Children

The notion that sensorimotor experience is essential to develop cognitive abilities is by no means a novel idea in the field of developmental psychology. The first developmental stage in Piaget’s (1936) pioneering theory of cognitive development was referred to as the sensorimotor stage. More recent research in infants confirmed the crucial impact sensorimotor interactions with the environment have on cognitive processes and their development (e.g., Adolph and Avolio, 2000; Thelen et al., 2001).

For instance, Smith (2005) examined the influence of motor actions on object recognition, and more specifically shape categorizations. Young children learned *new* associations between moving objects and their corresponding shapes. Results showed that performing an action but not watching an action altered the children’s associations of the action with the object, thereby providing evidence that building new associations, by means of sensorimotor interactions with objects, is crucial for the generation of conceptual knowledge about objects.

Next to object recognition, evidence from research in toddlers and infants suggests that new associations built through sensorimotor interactions modulate cognitive processes such as decision-making and choice selection (e.g., Rivière and Lécuyer, 2008; Rivière and David, 2013). Likewise, sensorimotor interactions are foundational for creating new higher-order representations (e.g., Boncoddo et al., 2010), language representations (Toumpaniari et al., 2015), and science concepts (Kontra et al., 2015).

Besides the evidence for embodied cognition effects driven by new associations during early childhood, there is also evidence for embodied cognition effects driven by the *reactivation* of previously generated associations. For instance, Frick and Moehring (2013) demonstrated that performance in mental rotation tasks was influenced by prior motor experiences. In their study, infants’ gazing times at both possible and impossible rotation events were measured and related to their previous locomotor experience, including rotational movement experience. The results showed that 10-month-old infants gazed longer at impossible rotation events than possible rotation events. An interaction between the observed rotation and prior locomotor experience indicated an experience-based influence of previous locomotor experience on mental rotation abilities.

In addition, there is also evidence for reactivation effects in the processing of language, namely the activation of motor areas of the brain when listening to action-related verbs as opposed to non-action-related adjectives (e.g., James and Maouene, 2009). Likewise, there is evidence that reactivation effects are present during visual-spatial and vocabulary tasks (Dellatolas et al., 2003), visual letter recognition (James, 2010), and number processing (Krinzinger et al., 2011) in children.

All in all, infants provide an important and unique testbed for embodied cognition effects, as they quickly grow through several developmental stages and make many pure and novel encounters with their environment (Daum et al., 2009). Embodied cognition research in young children provides evidence for embodied cognition effects driven by *new* associations (e.g., Smith, 2005; Boncoddo et al., 2010; Rivière and David, 2013) as well as embodied cognition effects driven by the *reactivation* of previously generated associations (e.g., Dellatolas et al., 2003; James, 2010; Frick and Moehring, 2013).

## Embodied Cognition Effects in the Elderly

The specific changes that occur in aging make the elderly particularly relevant for increasing our understanding of embodied cognition: on the one hand, cognitive functions decrease in the elderly and the sensorimotor system is not as flexible as in younger people which constrains the opportunities to build new associations (Calero and Navarro, 2007). On the other hand, although the individual differences in sensorimotor experiences should be taken into account, with increasing age the chance and likelihood to engage in any kind of interaction with the environment and hence generate sensorimotor experience increases. As a consequence, increasing age leads to an enriched possibility to access previously accumulated sensorimotor experience for cognitive processes.

To start with the former, an embodied cognition effect driven by *new* multimodal associations was tested by Erickson et al. (2011). Erickson et al. (2011) examined the impact of aerobic exercise on memory functions and the underlying brain structures in elderly participants. Results revealed that aerobic exercise training improved memory functions and increased the size of the hippocampus, indicating that age-related decreases of cognitive functions in the elderly can even be reversed by means of generating new sensorimotor experiences. These findings are supported by a study in an elderly participant group that suffered from Alzheimer’s disease (Bredesen, 2014). Significant life-style changes including novel physical activities and exercise improved participants’ cognitive functions. A meta-analysis by Scherder et al. (2014) confirmed that walking improved cognitive functions (i.e., set-shifting and inhibition) in healthy elderly people, who did not walk much in their daily life before the intervention period. As the studies report a change in cognitive functions due to a change in sensorimotor activities, they seem to verify the existence of embodied cognition effects as driven by new associations. Although we discuss these effects as being driven by new sensorimotor experience, it is certainly also possible that these effects are driven by *more* experience instead of *new* experience. Due to the fact that there is a paucity of research on this matter, caution in interpretation is warranted. Similarly, research on embodied cognition effects in elderly that reflect the influence of multimodal associations, that were previously built and are reactivated, is scarce.

A case in point for an embodied cognition effect driven by reactivated associations in healthy and memory-impaired elderly was reported by De Scalzi et al. (2015). De Scalzi et al. (2015) showed that elderly people made faster sensibility judgments on sentences when the direction of the described action was aligned with the response direction. This is referred to as the action compatibility effect. The existence of action compatibility effects in elderly with impaired short-term memory provides further evidence for embodied cognition effects driven by a *reactivation* of implicit associations previously learned or built. Next to evidence on the action compatibility effect in the elderly (Fernandino et al., 2013; De Scalzi et al., 2015), research on mental transformations of whole-body images also seems to confirm embodied cognition effects driven by reactivated associations (Conson et al., 2014).

Conclusively, only very few studies explicitly address embodied cognition effects in the elderly. It seems that the limited evidence supports the existence of embodied cognition effects driven by new and reactivated associations. However, a decisive conclusion about the specificity of embodied cognition effects in the elderly seems unwarranted given the lack of data. A currently more fruitful approach is to focus on comparing embodied cognition effects across different age groups including children, adults, and elderly people.

## Embodied Cognition Effects: A Comparison Across the Lifespan

In a study that looked at younger and older adults, Dijkstra et al. (2007) included both embodied cognition effects driven by new and reactivated associations. Participants held certain body postures and activated autobiographical memories that were congruent or incongruent to the body posture. Results indicated that congruent body postures facilitated the retrieval of autobiographical memories regardless of age. To examine if newly built associations might differ between age groups, 2 weeks after the experiment participants were asked to recall the postures they had executed during the manipulation. Results revealed an age-dependent difference; memory performance on new associations were better in younger than in older adults, suggesting that effects driven by new associations may be stronger in younger than in older adults.

Similar effects were reported for mental imagery performance. Frick et al. (2009) asked participants to tilt empty glasses, which were filled with different levels of imaginary water, until the water would be at the edge of spilling over. The tilting movement was executed with or without active motor control. Results showed that younger participants benefited more from the concurrent active motor execution than older participants, which suggests that younger participants relied on the novel sensorimotor association, whereas older participants relied more on reactivated knowledge to solve the task.

Another good indicator to examine whether embodied cognition effects are driven by the reactivation of previous associations is to test the ability to immediately construe word meanings during reading and to activate the stored sensorimotor experience associated with the representation. Madden and Dijkstra (2010) scrutinized how this automatic activation of representations develops with increasing age. They first presented participants with sentences and afterward showed a picture of the object that was named in the corresponding sentence. The shape of the object matched or mismatched the situation described in the sentence. Results showed an age effect in the way that the mismatch effect was smaller in younger than in older adults. Interestingly, this effect has been shown to be even smaller in children (Engelen et al., 2011), thereby providing further evidence for an increase of experienced-based embodied cognition effects that goes hand in hand with growing age.

Further evidence pointing into the same direction stems from laterality research. Casasanto and Henetz (2012) examined if the easier access to objects that are on the right side (for right handed participants) leads to a specific space-valence mapping in children, where right is positively associated and left negatively associated. Results showed that handedness was a valid predictor for the previously generated associations between valence and space, highlighting that children construct abstract concepts dependent on their handedness. Notably, this effect had previously been shown to be even stronger in adults (Casasanto, 2011). In a similar vein, experience-based embodied cognition effects of body-object interactions were shown to also increase with age (Wellsby and Pexman, 2014).

The comparison of embodied cognition effects between different age groups including children and elderly revealed that age has a significant impact on embodied cognition effects and their underlying processes (e.g., Dijkstra et al., 2007; Frick et al., 2009). Whereas embodied cognition effects driven by new associations seem to be stronger in younger than in elderly people (Dijkstra et al., 2007; Frick et al., 2009), embodied cognition effects driven by the reactivation of previously built associations tend to be stronger with increasing age (e.g., Engelen et al., 2011; Dekker et al., 2014). This empirical difference clearly shows that findings from the adult population are obviously not representative for the whole age spectrum.

## Discussion and Conclusion

We submit that a lifespan approach is mandatory to take significant steps toward a unifying framework of embodied cognition. We classified the underlying processes of embodied cognition effects as either being driven by *new* associations or *reactivated* associations. Our assessment revealed that both types, i.e., new and reactivated associations, are prevalent at both extremes of the lifespan. Embodied cognition effects driven by new associations seem to be stronger in the younger population than in the elderly. By contrast, embodied cognition effects that are driven by the reactivation of previously built associations tend to be stronger with increasing age. These findings question the representativeness of current theories across the whole age spectrum and underpin the necessity for a lifespan approach toward embodied cognition. Moreover, our findings bear significant consequences for a multitude of applied settings such as educational settings (e.g., the optimization of learning processes in school children) or working with elderly in retirement homes (e.g., the maintaining or regaining of cognitive capacities through sensorimotor and rehabilitation training).

We advocate that future research should focus on systematically investigating embodied cognition over the lifespan both on a theoretical level by testing age-specified hypotheses as well as on a methodological level by applying appropriate, age-based designs. To start with theory, the first hypothesis that needs to be scrutinized is that effects driven by new associations *decrease* with age and effects driven by the reactivated associations *increase* with age. Given that sensorimotor and cognitive capacities and their interactions are more fragile at both extremes of the lifespan, the second hypothesis that needs to be tested is that this fragility may correspond with a *higher inter-individual variance* in children and the elderly when compared to adults (**Table 1**). The hypotheses in **Table 1** are meant to be a starting point for empirical tests, falsification and the generation of alternative hypotheses. Although we argue for a higher inter-individual variance at the extremes of the lifespan when compared to adults due to fragile and, therefore, diverging sensorimotor and cognitive abilities between individuals, one could equally predict that increased variability in sensorimotor processes could lead to increases in, both, inter-individual and intra-individual variability. For instance, it is feasible that fragile sensorimotor and cognitive capacities, and their interactions at both extremes of the lifespan, lead to increases in inter-individual and intra-individual variability in task performance, if the task recruited such processes.

**Table 1:** Classification and hypotheses for embodied cognition effects across the lifespan.

	New/reactivated associations	Intra-/inter-individual variance
Young	New>reactivated	Inter>intra
Middle	New=reactivated	Intra=inter
Old	New<reactivated	Inter>intra


The theoretical consequences of incorporating age as an essential variable in the formation of embodied cognition theories at the same time bear important consequences for the methodological choices. Our methodological recommendations are threefold:

First, we strongly suggest that future studies on embodied cognition should – next to cross-sectional studies – include longitudinal designs. Disentangling new versus reactivated sensorimotor experiences and their impact on cognitive processes, in our view, is one of the biggest methodological challenges with respect to a lifespan approach.

Second, in particular for cross-sectional studies, an important approach may reside in simulating physical conditions that are to the same extent unfamiliar to different age groups in order to diminish the influence of former motor experience (e.g., absence of gravity, age simulation suits).

Third, age-based tests that reliably reflect the mutual influence of sensorimotor and cognitive processes at different age stages need be developed. To stick with the introductory example of problem solving: to scrutinize the effect of age on the embodied processes underlying problem-solving tasks, the development of problem-solving tasks that are objective, reliable, and valid across different age groups is required. This is challenging, as the respective tasks may need to be adjusted to the faced age-constraints.

To summarize, adopting a lifespan approach toward embodied cognition is mandatory and challenging at the same time. Here we highlighted the necessity and offered a classification that may help to systematically apply a lifespan approach. Finally, we proposed falsifiable hypotheses and gave methodological advise on how to test these with the intention to further our understanding of embodied cognition and to spark new research avenues that may also enrich the applied value of embodied cognition research.

## Author Contributions

All authors listed, have made substantial, direct and intellectual contribution to the work, and approved it for publication.

## Conflict of Interest Statement

The authors declare that the research was conducted in the absence of any commercial or financial relationships that could be construed as a potential conflict of interest.
